# Acute Marjolin’s Ulcer in Chronic Foot Wounds with Previous Negative Biopsy: Report of Two Cases

**DOI:** 10.5704/MOJ.2011.033

**Published:** 2020-11

**Authors:** S Xu, YBJ Kang, H Soeharno, NEM Yeo

**Affiliations:** Department of Orthopaedic Surgery, Singapore General Hospital, Singapore

**Keywords:** Marjolin's ulcer, acute, malignant transformation, case report

## Abstract

Marjolin’s ulcer is a rare and often overlooked diagnosis which can be encountered by a variety of specialities. Majority of the literatures describe long latency period of 11 to 75 years. The authors present two unusual cases of rapid progression to Marjolin’s ulcer in patients with previously negative biopsy 8- and 10-month prior. This highlights the importance for clinicians to have a high degree of suspicion when encountered with any non-healing wound, especially one who’s symptomatology and morphological features have undergone an acute change. Even with previous negative biopsy, patients should still be followed-up closely and clinicians should not hesitate to perform re-biopsy if the suspicion arises.

## Introduction

The term “Marjolin’s ulcer” was first coined by John Chalmers DaCosta in 1903 when he documented two cases of chronic varicose ulcers undergoing carcinomatous change, describing the malignant degeneration of skin scars1. Since then, it has been used to describe malignant tumours arising in chronic wounds, particularly burn scars. Marjolin’s ulcers are rarely seen as a prolonged latent period is necessary for malignant change. Acute malignant transformation is rare and present literature describes this latency to be between 11 to 75 years, with the average onset of disease during the 5th decade of life^2,3^. Biopsy is the gold standard for diagnosis of Marjolin’s ulcer with a false negative rate of less than 2% when performed accurately.

The authors present two unusual cases of patients who developed Marjolin’s ulcer despite having negative biopsies few months prior to highlight the need for a high degree of suspicion when treating any chronic non-healing wound, even with previous negative biopsy.

## Case Report

### Case 1

**Fig. 1:**
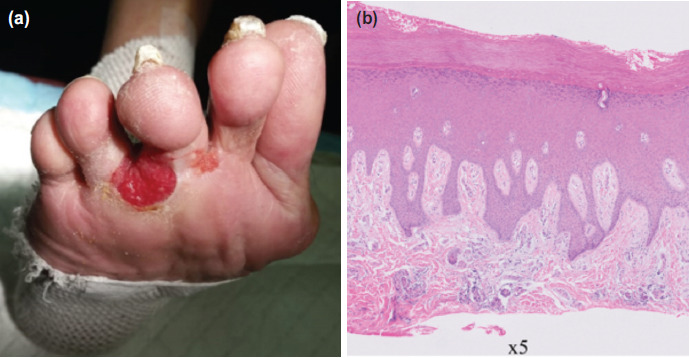
(a) Case 1 on initial presentation showing a superficial ulcer over the plantar aspect of the 3rd and 4th toe web space. (b) Histology section showing acanthosis with marked compact orthokeratin, in keeping with acral location, no dysplasia or malignancy seen.

The patient is an 87-year-old Chinese male who first developed a superficial ulcer over the plantar aspect of his third and fourth toe web space from scratching three years ago (Fig. 1a). Biopsy performed in August 2017 revealed epidermal acanthosis with no evidence of dysplasia (Fig. 1b). Tissue cultures grew mixed bacteria and he was treated with oral antibiotics and discharged with outpatient dressing change.

**Fig. 2:**
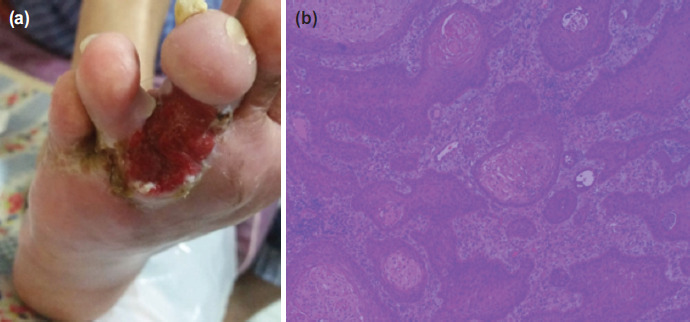
(a) Case 1 on second presentation showing a large fungating ulcer measuring 3 x 2cm over the plantar aspect of the third and fourth interdigital space. (b) Histology section showing extensive squamous cell carcinoma in situ with no evidence of co-existing melanoma or basal cell carcinoma.

The patient subsequently presented to Orthopaedic Surgery in May 2018 (eight months after initial presentation) with sudden increase in size of ulcer over five months associated with pain. A large fungating ulcer measuring 3 x 2cm over the plantar aspect of the third and fourth interdigital space was noted (Fig. 2a).

MRI revealed an enhancing soft tissue mass in the plantar aspect of the forefoot. Biopsy was performed and histology revealed features consistent with well-differentiated squamous cell carcinoma. He subsequently underwent a right forefoot amputation, successfully obtaining 2cm clear margin, with intra-operative frozen sections negative for malignancy. The final histology revealed extensive squamous cell carcinoma-in-situ (Fig. 2b). Patient has since recovered well with no signs of local recurrence at two years three month follow-up.

### Case 2

The patient is a 68-year-old Chinese male who first presented in January 2018 with a superficial ulcer over the sole of his right forefoot for 1-year. Biopsy revealed reactive epidermal hyperplasia negative for dysplasia or malignancy. Tissue culture showed fungal hyphae and he was treated with oral antibiotics and topical antifungal.

**Fig. 3:**
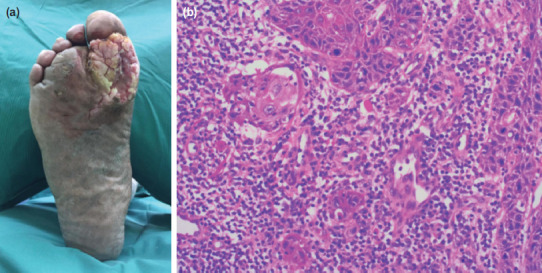
(a) Case 2 on secondary presentation showing a large 7 x 5cm exophytic ulcer over the base of the first metatarsal-phalangeal joint (MTPJ) extending into first and second webspace. (b) Histology section showing infiltrating nests and islands of well differentiated squamous cell carcinoma, accompanied by inflammation.

The patient presented to Orthopaedic Surgery in Nov 2018 (10-month after initial presentation) with an enlarging lesion of his forefoot. A large 7 x 5cm exophytic ulcer was noted over the base of the first metatarsal-phalangeal joint (MTPJ) extending into first and second webspace (Fig. 3a).

MRI revealed plantar ulcer over first MTPJ with exophytic soft tissue lesion in first and second webspace. Excision biopsy revealed well-differentiated squamous cell carcinoma. The patient subsequent underwent a right forefoot amputation. Final histology was reported as well-differentiated squamous cell carcinoma (Fig. 3b). Patient recovered well with no signs of local recurrence at one year seven month follow-up.

## Discussion

The prolonged latent period and seemingly benign precursor lesions which this squamous cell carcinoma arises from often results in clinicians overlooking the possibility of a malignant process. Various pathogenic pathways have been postulated for the development of Marjolin’s ulcer. Scar tissue is inherently less resistant to mechanical trauma and heals with difficulty, especially when located in the joint area. It has been hypothesised that cells forming the scar tissue releases toxins via autolysis and heterolysis. Together with the polymicrobial infection present in many ulcers, healing of such chronic wounds is prolonged and leaves groups of rapidly regenerating cells susceptible to the formation of mutagens. Blood and lymphatic vessels also regenerate poorly and scar tissue becomes immunologically deficient, causing malignant cells to escape immune mediated elimination^4^.

Latency is defined as the time between diagnosis of the primary pathology and the histopathological confirmation of a Marjolin’s ulcer. It has been shown that this is inversely proportional to patient’s age. The largest systemic review in literature by Kowal-Vern *et al*^2^ of 412 cases found the mean age of diagnosis to be 50 years with a latency period of 30 years.

The authors present two unusual cases of rapid progression to Marjolin’s ulcer in patients with previously negative biopsy 8 and 10 months prior. The presence of bacteria growth pointed towards a diagnosis of infection. This further highlights the need for a high degree of suspicion when reviewing patients with chronic non-healing wounds. Patient should be followed-up regularly and given advice to return if wound does not heal despite appropriate antibiotics and wound treatment. Clinicians should also not hesitate to perform re-biopsies when there is any suspicion of disease progression.

Patients who present with chronic untreated wounds often come from socio-economically poorer background, thus compliance to treatment and follow-up might be a challenge. Therefore, in this group, patient education and reinforcing compliance to regular follow-up and treatment is particularly important so as not to miss a Marjolin’s ulcer.

The most common histology of Marjolin’s ulcer is squamous cell carcinoma (~71%), followed by basal cell carcinoma (~12%) and malignant melanoma (~6%)2. However, in cases of acute transformations, the ulcer is most often a basal cell carcinoma arising from burn scar. There is a paucity of literature on acute malignant transformation to squamous cell carcinoma. A case series of three patients by Chang *et al*^3^ describes one case of acute malignant transformation of a burn wound to squamous cell carcinoma in a 60-year-old man within three months. Mohammadi *et al*^5^ also reported one case of a man who developed acute Marjolin’s ulcer six weeks after a burn injury. In this case report, the authors described two acute malignant transformation of a chronic traumatic wound to squamous cell carcinoma which is one that has not been well described in literature.

One important consideration in diagnosis of Marjolin’s ulcer is the possibility of false negative results from biopsy due to sampling error. The gold standard in diagnosis of Marjolin’s ulcer is biopsy and if adequate samples from different parts of the wound had been taken, biopsy is usually accurate with a false negative rate of less than 2%. The authors recommend that multiple incision or wedge biopsies be taken from different part of the wounds to minimise the false negative rate. In cases of true negative biopsy, patients should still be closely followed-up and re-biopsies taken if wound continues to be non-healing.

In conclusion, these two cases highlight the importance for clinicians to have a high degree of suspicion when treating any non-healing wound, particularly one who’s symptomatology and morphological features have undergone acute changes. The authors propose that a patient’s risk of developing a Marjolin’s ulcer should be determined by individual patient factors that might shorten the latency of malignant transformation. As such, elderly patients, or those with chronic wounds arising from burn injuries should be closely followed-up, with lower thresholds for carrying out re-biopsies to obtain accurate and early histological diagnoses of suspicious lesions.
